# Supplementary material for: ‘Ghost’ fossils of early coccolithophores point to a Triassic diversification of marine calcifying organisms
Source: Nat Commun. 2025 Oct 20;16:9283. doi: 10.1038/s41467-025-65116-0 (PMC12537907; doi:10.1038/s41467-025-65116-0)
Supplement: Supplementary file 1 — Supplementary Information [file 41467_2025_65116_MOESM1_ESM.pdf]

Supplementary Information

‘Ghost’ fossils of early coccolithophores point to a Triassic diversification of marine calcifying organisms

Sam M. Slater[1*], Isaline Demangel[1,2] & Sylvain Richoz[2]

[1]Department of Palaeobiology, Swedish Museum of Natural History, Stockholm, Sweden. [2]Department of Geology, University of Lund, Lund, Sweden. e-mail: sam.slater@nrm.se

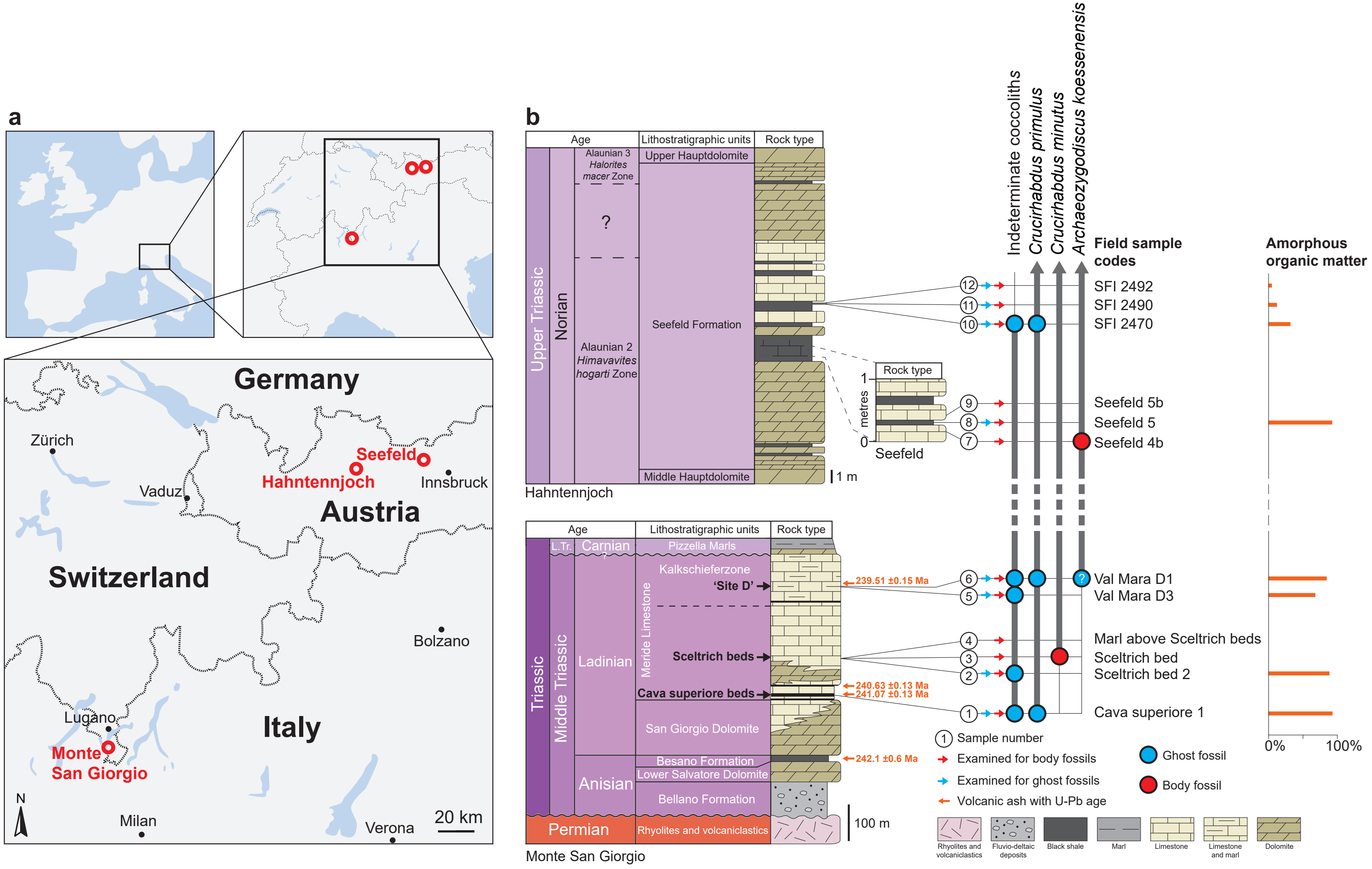

Supplementary Fig. 1. Study sites and fossil occurrences. **a,** Study site locations. **b,** Stratigraphic context of sampled localities, age of samples with ages of volcanic ash layers, coccolith fossil occurrences and amorphous organic matter relative abundances. Part (a) modified from[1]; stratigraphy of Monte San Giorgio in (b) modified from[2,3].

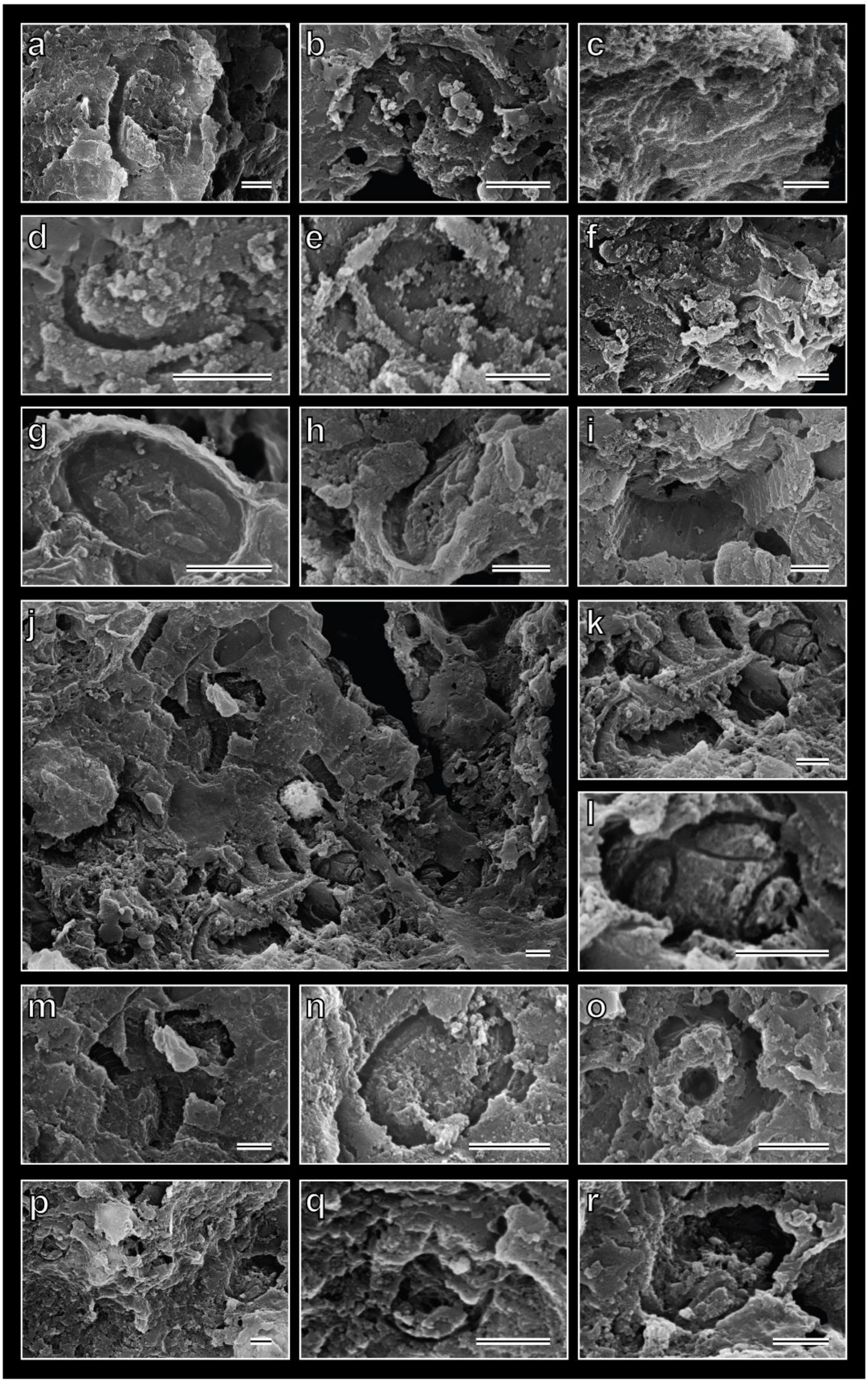

Supplementary Fig. 2. Additional Ladinian coccolith 'ghost' fossils.
a, *Crucirhabdus primulus*, sample 1 (Cava superiore 1; S206000-03). **b,** *C. primulus*, sample 1 (Cava superiore 1; S206000-03). **c,** Indeterminate coccolith, sample 2 (Sceltrich bed 2; S206001-03). **d, e,** Indeterminate coccoliths, sample 5 (Val Mara D3(ii); S206002-05). **f,** Several *C. primulus*, sample 6 (Val Mara D1; S206003-03). **g,** *C. primulus*, sample 6 (Val Mara D1(ii); S206003-05). **h,** *C.*

primulus, sample 6 (Val Mara D1; S206003-03). **i,** *C. primulus*, sample 6 (Val Mara D1(ii); S206003-05). **j,** Several *C. primulus*, sample 6 (Val Mara D1; S206003-03). **k–m,** Enlarged image of **j**. **n, o,** *C. primulus*, sample 6 (Val Mara D1(iii); S206003-06). **p,** Several *C. primulus*, sample 6 (Val Mara D1; S206003-03). **q, r,** Enlarged image of **p**. Scale bars, 1 µm.

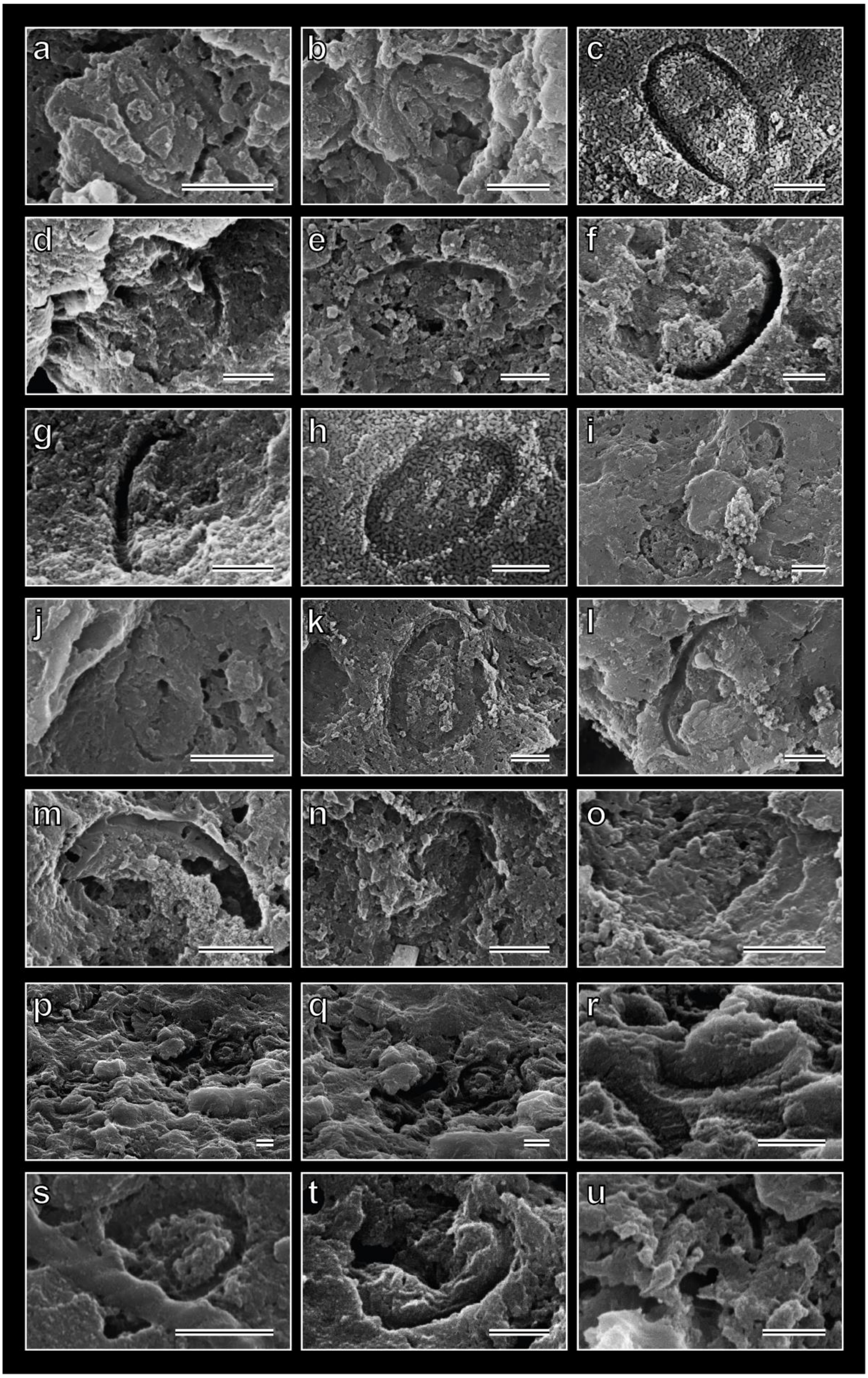

Supplementary Fig. 3. Additional Ladinian and Norian coccolith 'ghost' fossils. a, b, *Crucirhabdus primulus*, sample 6 (Val Mara D1(ii); S206003-05). **c,** Indeterminate coccolith, sample 6 (Val Mara D1; S206003-03). **d–h,** Indeterminate coccoliths, sample 6 (Val Mara D1; S206003-03). **i–k,**

Indeterminate coccoliths, sample 6 (Val Mara D1(iii); S206003-06). **l–o,** Indeterminate coccolith, sample 6 (Val Mara D1(ii); S206003-05). **p,** Several *C. primulus*, sample 10 (SFI 2470; S206005-03). **q, r,** Enlarged image of **p**. **s, t,** *C. primulus*, sample 10 (SFI 2470; S206005-03). **u,** Indeterminate coccolith, sample 10 (SFI 2470; S206005-03). Scale bars, 1 µm.

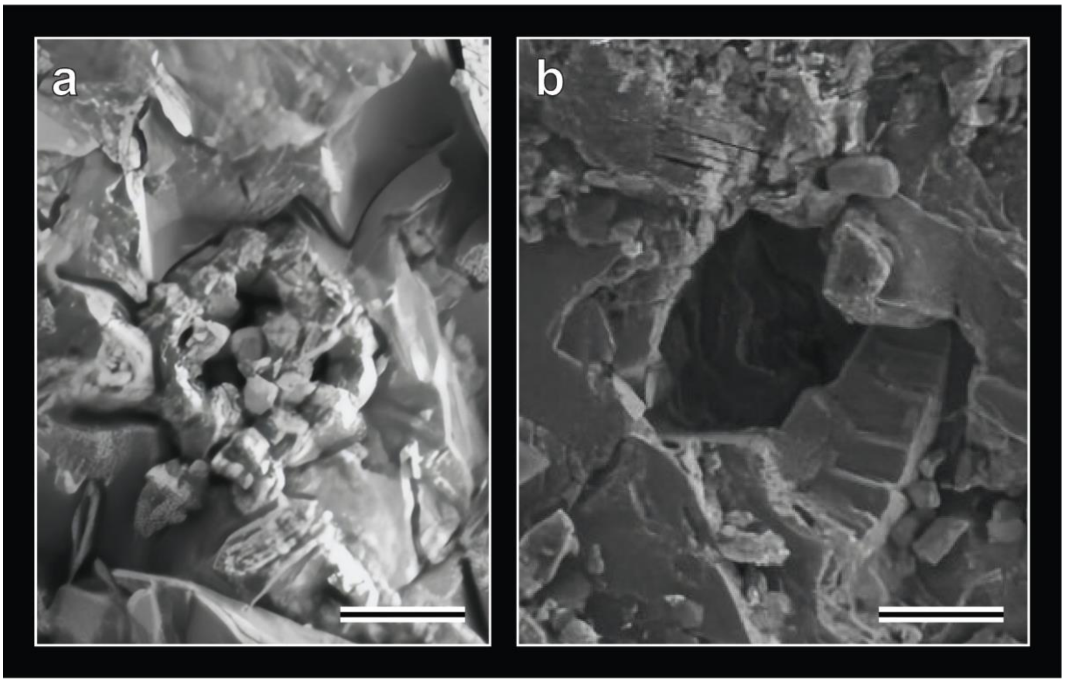

Supplementary Fig. 4. Coccolith 'body' fossils. a, *Crucirhabdus minutus*, sample 3 (Sceltrich bed; S206008-01). **b,** *Archaeozygodiscus koessenensis*, sample 7 (Seefeld 4b; S206010-01). Scale bars, 1 µm.

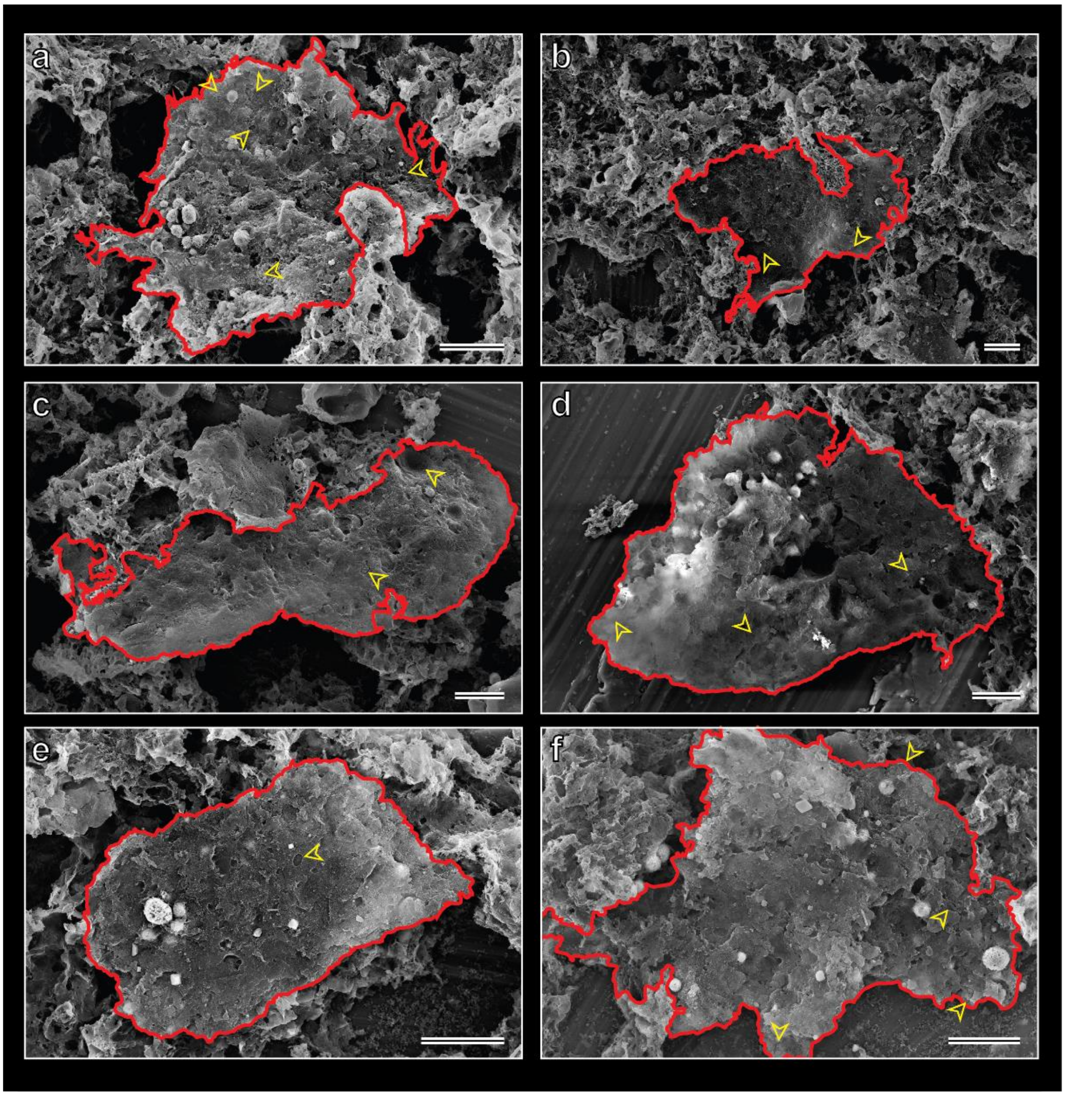

Supplementary Fig. 5. Various types of amorphous organic matter (AOM). a–d, Sample 6 (Val Mara D1; S206003-03). **e, f,** Sample 6; (Val Mara D1(ii); S206003-05). Smooth AOM outlined in red, surrounded by background angular AOM. Arrows mark coccolith 'ghost' fossils. Scale bars, 20 µm.

Supplementary Note 1

All samples processed for palynofacies analysis (1, 2, 5, 6, 8, 10–12) yielded organic matter (see Supplementary Data 3 for raw data). Ladinian samples (1, 2, 5, 6) were overwhelmingly dominated by amorphous organic matter (AOM). Most Norian samples (10–12) were dominated by translucent and opaque phytodebris (including wood and charcoal), except for sample 8, which similarly to Ladinian samples, was also dominated by AOM (Supplementary Fig. 1 and Supplementary Data 3). Samples 11 and 12 yielded the lowest abundances of AOM. These, along with sample 8, were the only samples processed for organic matter that were barren of coccolith ghost fossils. The absence of ghost fossils in samples 11 and 12 reflects a lack of AOM onto which coccoliths can imprint. Sample 8 appears anomalous, as this yielded abundant AOM, but no ghost fossils. This is likely due to the type of AOM preserved; although AOM may be abundant, ghost fossils here were only found on a rare type of smooth AOM (Supplementary Fig. 5), which was not observed in sample 8 when viewed using a scanning electron microscope. These findings demonstrate that both the abundance and type of AOM are equally important factors in determining the preservation potential of coccoliths as ghost fossils.

References in Supplementary Information

1. Stockar, R., Baumgartner, P. O. & Condon, D. Integrated Ladinian bio-chronostratigraphy and geochronology of Monte San Giorgio (Southern Alps, Switzerland). *Swiss Journal of Geosciences* **105**, 85–108 (2012).
2. Stockar, R., Adatte, T., Baumgartner, P. O. & Föllmi, K. B. Palaeoenvironmental significance of organic facies and stable isotope signatures: The Ladinian San Giorgio Dolomite and Meride Limestone of Monte San Giorgio (Switzerland, WHL UNESCO). *Sedimentology* **60**, 239–269 (2013).
3. Montagna, M., Magoga, G., Stockar, R. & Magnani, F. The contribution of the Middle Triassic fossil assemblage of Monte San Giorgio to insect evolution. *Communications Biology* **7**, 1023 (2024).
4. Hopf, H., Thiel, V. & Reitner, J. An example for black shale development on a carbonate platform (Late Triassic, Seefeld, Austria). *Facies* **45**, 203–210 (2001).
5. Ogg, J. G., Chen, Z.-Q., Orchard, M. J. & Jiang, H. S. The Triassic Period. In *Geologic Time Scale 2020* (eds Gradstein, F. M., Ogg, J. G., Schmitz, M. D. & Ogg, G. M.) 903–953 (Elsevier, 2020).
6. Donofrio, D. A., Brandner, R. & Poleschinski, W. Conodonten der Seefeld-Formation: Ein Beitrag zur Bio- und Lithostratigraphie der Hauptdolomit-Plattform (Obertrias, westliche Nördliche Kalkalpen, Tirol). *Geologisch-Paläontologische Mitteilungen* **26**, 91–107 (2003).
7. Galbrun, B. et al. "Short" or "long" Rhaetian? Astronomical calibration of Austrian key sections. *Global and Planetary Change* **192**, 103253 (2020).

8. Demangel, I. et al. Development of early calcareous nannoplankton in the Late Triassic (Northern Calcareous Alps, Austria). *Global and Planetary Change* **193**, 103254 (2020).
9. Janofske, D. Calcareous nannofossils of the Alpine Upper Triassic. In *Nannoplankton Research, Proceedings of the 4th INA Conference, Prague 1991* Vol. 1 (eds Hamrsmid, B. & Young, J. R.), *Knihovnicka ZPN* **14a**, 87–109 (1992).
10. Jafar, S. A. Significance of Late Triassic calcareous nannoplankton from Austria and Southern Germany. *Neues Jahrbuch für Geologie und Paläontologie* **166**, 218–259 (1983).
11. Payne, J. L. et al. Large perturbations of the carbon cycle during recovery from the end-Permian extinction. *Science* **305**, 506–509 (2004).
