# Supplementary material for: ‘Ghost’ fossils of early coccolithophores point to a Triassic diversification of marine calcifying organisms
Source: Nat Commun. 2025 Oct 20;16:9283. doi: 10.1038/s41467-025-65116-0 (PMC12537907; doi:10.1038/s41467-025-65116-0)
Supplement: Supplementary file 2 — Description of Additional Supplementary Files [file 41467_2025_65116_MOESM2_ESM.pdf]

Description of Additional Supplementary Files

Supplementary Data 1. Sample numbers, field codes, locations and stratigraphic details of all samples.

Supplementary Data 2. Age ranges of various species and groups featured in Fig. 4. We follow Ogg et al. (2020)[5] for Triassic stage boundary age estimates. For the placing of the Norian/Rhaetian boundary, we follow the age estimate from the Austrian GSSP candidate section (Steinbergkogel). This is because several of the previous initial occurrences of numerous coccolith taxa have been recorded through that section[7], and thus these previous initial occurrences are internally consistent with the placement of the Norian/Rhaetian boundary at Steinbergkogel.

Supplementary Data 3. Count data of various organic matter (palynofacies analysis) categories for each sample.

Supplementary Movie 1. Coccolith 'ghost' fossil preserved on amorphous organic matter, sample 6 (Val Mara D1(ii); S206003-05). Note that the final frame is an inverted image of the ghost fossil.
